# The Effect of Caffeine Supplementation on Female Volleyball Players’ Performance and Wellness during a Regular Training Week

**DOI:** 10.3390/nu16010029

**Published:** 2023-12-21

**Authors:** Jesús Siquier-Coll, Gabriel Delgado-García, Fulgencio Soto-Méndez, Antonio Liñán-González, Raquel García, Francisco Tomás González-Fernández

**Affiliations:** 1 Department of Communication and Education, Universidad Loyola Andalucía, 41704 Dos Hermanas, Spain; 2 SER Research Group, Department of Physical Activity and Sport Sciences, Center of Higher Education Alberta Giménez, Affiliated to Pontifical University of Comillas, 07013 Palma, Spain; gadelgado@cesag.comillas.edu (G.D.-G.); fsoto@cesag.org (F.S.-M.); 3 Department of Nursing, Faculty of Health Sciences, Melilla Campus, University of Granada, 52005 Melilla, Spain; 4 Department of Nursing, Faculty of Health Sciences, University of Granada, 18071 Granada, Spain; raquelgp@ugr.es; 5 Department of Physical Education and Sports, Faculty of Sport Sciences, University of Granada, 18071 Granada, Spain; ftgonzalez@ugr.es

**Keywords:** supplementation, caffeine, sport performance, volleyball, ergogenic aids

## Abstract

Background: caffeine is an ergogenic aid that still needs to be investigated in women’s sports performance. Methods: Eight semi-professional women’s volleyball players (height = 1.63 ± 0.08 m; weight = 66.67 ± 4.74 kg) voluntarily participated in this study. A randomized crossover design was implemented where players underwent caffeine and placebo conditions. In the caffeine condition, participants consumed 5 mg/kg of caffeine based on their body weight before acute training. The evaluations were performed over two weeks of training. In both conditions, the countermovement jump, repeated jumps for 15 s, and handgrip tests were performed. The change of direction was assessed using the 505 test. Well-being was also assessed with a wellness questionnaire. A repeated measures ANOVA and correlation analysis were performed. Results: The repeated measures ANOVA revealed a main effect of supplementation (*F* (1.7) = 8.41, *p* = 0.02, η2 = 0.54) across the training week on physical performance. Additionally, there was a positive effect on perceived fatigue (*F* (1.7) = 7.29, *p* = 0.03, η2 = 0.51). Conclusions: Caffeine improved performance and fatigue parameters over one week of training. Further research is needed on women, focusing on physical performance and wellbeing, especially during intense periods.

## 1. Introduction

High performance exposes athletes to an exacting physical and mental load due to competition and training. These demands lead athletes to turn to ergogenic aids as a strategy to maintain performance and combat fatigue. Volleyball, one of the most popular sports in the world [1], involves specific tasks such as jumping, landing, blocking, and throwing the ball, which in turn must be combined with fast movements. Hence, it makes high demands on the musculoskeletal system [2].

The use of caffeine is considered a potential ergogenic aid, able to enhance the athletic performance of volleyball players [3]. It is widely used as an ergogenic aid in both individual and team sports due to its fast, stimulating effect [4]. In addition, it has been classified as a safe supplement by the International Society of Sports Nutrition (ISSN) [5]. The use of this ergogenic aid is on the rise due to its effect on aerobic [6,7] and anaerobic activities [3,6,8], increasing strength and power capacity [9] by enhancing intracellular calcium and Na^+^-K^+^ ATPase pump activity [10], and delaying the onset of fatigue [11] through activation of the central nervous system, which blocks the adenosine receptors [11,12,13].

Several studies have reported the effect of caffeine on jumping improvement [3]; nevertheless, the effect on agility tests, such as changes of direction, is unclear. Some studies suggest that it does not improve them [8], while another study on women’s volleyball players describes a significant positive effect [14]. More evidence is needed to determine the effect of caffeine on agility, especially in women’s volleyball. Gomez-Bruton et al. (2021) conclude that acute caffeine intake is capable of enhancing team sports performance in female athletes. Therefore, it could be effective as an ergogenic aid for female team athletes [15]. Regarding dose intake, it is well established that a range from 3 to 9 mg/Kg enhances athletic performance [6,8,16,17]. Concerning the timing of ingestion, caffeine intake one hour before a training session has been shown to be an optimal strategy to enhance performance [18] due to its fast absorption and plasma availability [11].

Volleyball requires weekly competition, and proper load control must be managed to balance the stress-recovery cycle and maintain high performance throughout the season [19,20]. Recently, the term internal load has been introduced into team sports, highlighting the importance of monitoring the fatigue and stress induced by competitions, training sessions, and daily life since it is a determinant, along with the external load, of training outcome [21,22,23]. Accordingly, subjective wellness questionnaires are suggested as convenient instruments for measuring internal load in team sports athletes [21,24,25]. The questionnaires reflect players’ perceptions of muscle pain [26], general fatigue [25], sleep quality [27], rating of perceived exertion [28], and psychological stress [29].

Caffeine has been proven to deliver positive outcomes in reducing the rating of perceived exertion [30,31,32,33], diminishing muscular soreness or damage [34,35], although to a lesser degree than in men [30,33], and enhancing performance in the eumenorrheic population [4,10,36]. However, a main undesirable aspect to consider regarding caffeine supplementation in athletes is that it could negatively affect sleep quality [37], especially in women athletes because the effect of caffeine persists longer in women than in men [38]. Recent studies have highlighted the lack of research on the caffeine dose–response with factors including sleep, fatigue, and performance assessments [39,40]. Indeed, recent research reported that there are still more studies on men, with few investigations on women [18], specifically regarding strength [41]. Thus, further studies are required on the effect of caffeine on strength in women. In addition, the scientific literature on the ergogenic effect of caffeine on women’s volleyball players is scarce or lacking, which highlights the importance of this topic in volleyball. Hence, it is important to know its effects on physical performance, fatigue, and wellness. Therefore, the purpose of this study was to establish the ergogenic effect of 5 mg/kg caffeine on wellness (sleep, fatigue, stress, and muscle soreness) and physical performance (COD 505 test, CMJ height, RJ height, RJ RSI, RJ min jump, RJ max jump, RJ fat index, and RJ time count) in women’s volleyball team athletes.

## 2. Materials and Methods

### 2.1. Experimental Design

**Figure 1:**
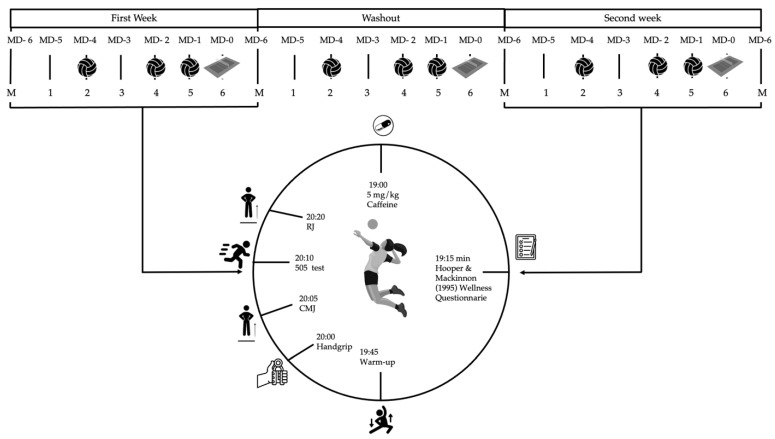
Experimental design. MD: Match Day; CMJ: Countermovement Jump; and RJ: Repeated Jump. Consult ‘Procedure’ section for further information [42].

**Figure 2:**
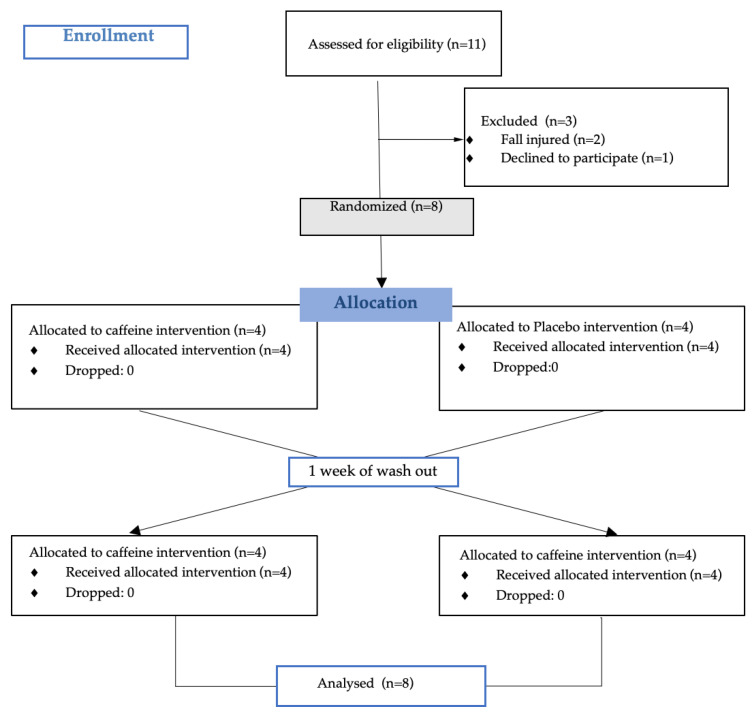
Consort flow diagram.

The present study was conducted in March 2022, at which point the semi-professional women’s volleyball players had completed six months of training. A randomized, double-blind, crossover design was used, in which each participant underwent the placebo condition for one week and the caffeine condition for another week. Participants rotated between the placebo condition and the caffeine condition; while half of the participants started with the caffeine condition, the other half of the participants started the study with the placebo condition. After one week of washouts, the conditions alternated. During the caffeine condition, the participants consumed 5 mg/kg body weight of caffeine anhydrous powder (www.hsnstore.com; accessed on 31 January 2022) mixed with a maltodextrin-based beverage one hour before the measurements, while during the placebo condition, they ingested only the maltodextrin-based beverage. Both beverages were ingested one hour before the measurements. The dose and timing of intake were established following previous studies carried out on male volleyball players [3]. The players filled out the Hooper & Mackinnon (1995) [42] wellness questionnaire one hour before training. González-Fernández et al. (2022) [43] can be consulted for further information. The physical measurements took place on the volleyball court just before training (Tuesday—MD-4, Thursday—MD-3, and Friday—MD-1) at 8:00 p.m. The players performed a warm-up similar to the match warm-up. Afterwards, they performed the measurements in the following order: handgrip, CMJ, 505 test, and RJ15”. For each of the measurements, two attempts were made, separated by 3 min, and the better score was taken, excluding RJ15”, for which only one set was assessed. Between each of the tests, there was a 3 min recovery time. Before starting session 1, anthropometric data were measured. See Figure 1 (experimental design) and Figure 2 (consort flow diagram) for more information.

### 2.2. Participants

A sample of eight semi-professional women’s volleyball players from the “Spanish Women’s Superleague 2” between the ages of seventeen and twenty-five (height: 1.63 ± 0.08 m; weight: 66.67 ± 4.74 kg; fat mass: 22.32 ± 2.50%; muscle mass: 50.26 ± 3.56 kg; bone mass: 2.70 ± 0.18 kg; body mass index: 19.3 ± 1.45; body water: 57.37 ± 1.58, and V02max: 41.77 ± 1.67) voluntarily participated in this study. The semi-professional women’s volleyball players performed a total of three days of training per week (90 min per training session) and played one match a week. An initial nutritional survey was carried out through the MyFitnessPal^®^ [44] application to determine the macronutrient intake (carbohydrates: 232.16 ± 45.62; fats: 74.33 ± 11.87; proteins: 92.15 ± 25.45; and total Kcal: 1966.34 ± 317.75). A priori sample size calculation was performed using a free online tool, G*Power (www.gpower.hhu.de; accessed on 2 February 2022), with a power level of 95% and an α _level of 0.05 and based on previous and similar studies [45], and it revealed that the sample size of >5 would be sufficient for the analysis.

Inclusion criteria for the participants in this study were (i) to be a player with at least 5 years of experience, (ii) not to have been taking supplementation or medication at least two weeks before the start of the study, (iii) not to have taken contraceptives and not to have suffered an injury in the previous 6 months, (iv) to have given their informed consent, (v) to have a regular caffeine intake lower than 100 mg/day, (vi) not to have any chronic pathology, (vii) not to change nutritional habits, (viii) being a eumenorrheic woman, and (ix) to be an active player with a federation license and participate in all training sessions during the intervention program.

This study was reviewed and approved by the Bioethics Committee of the University of Granada (registration number: 3014/CEIH/2022; date: 25 January 2023). This research was conducted under the guidelines of the World Helsinki Assembly, updated in Fortaleza in 2013 at the World Medical Assembly, for the study of human subjects. All participants were advised not to take drugs or medications before or during this study and to maintain their usual dietary habits. They were also instructed not to take any supplements for at least 2 weeks prior to the study. Specifically, they were provided with a list of the most common caffeine-rich beverages so that they would not be consumed during this period. The phases of the menstrual cycle were calculated from the record of the last menstrual period. In the supplementation condition, five players were in the luteal phase and three in the follicular phase of the menstrual cycle, while four were in the luteal phase and four in the follicular phase of the menstrual cycle in the placebo condition.

### 2.3. Procedure

Familiarization: First, the team staff was informed about the objectives of the study, and the research team ensured that the parents and participants signed their informed consent after having received details of the possible benefits and risks of the study. Subsequently, the research team studied and planned the structure of training together with the coaches.

Secondly, the semi-professional women’s volleyball players underwent a familiarization session with the tests (handgrip, CMJ, COD 505 test, and RJ). It is important to note that the tests followed the same order, with a minimum of 3 min rest between them.

The anthropometry and all tests were performed on a volleyball court with an average temperature of 11–17 °C and 60–80% relative humidity (stable temperature of 14 °C and relative humidity of 72%). Subsequently, the Yo-Yo intermittent recovery test—Level 1 (YYIRT1) was performed. This test was developed only in a pretest on the maximal oxygen uptake (VO2max in mL/min/kg). This parameter was calculated using the following equation: Yo-Yo IR1 test: VO2max (in milliliters per minute per kilogram) = IR1 distance (in meters) × 0.0084 + 36.4 [46].

The tests used in this research are described as follows:

Anthropometric characteristics: First, body composition was evaluated before the training session (09:00 p.m.) with participants in shorts and having removed shoes and any metal and jewelry prior to assessment. For the evaluation of body composition, the Bioelectrical Impedance Analysis (BIA) method was used with a TANITA^®^ (MC980MA PLUS, Arlington Heights, IL, USA).

Countermovement jump (CMJ): The CMJ was evaluated using the Chronojump-Boscosystem^®^ (Barcelona, Spain) (version 2.0.2.) that presents an intraclass correlation between 0.821 and 0.949 to measure jump height. This system was connected to a Microsoft Windows Computer (w.11). Participants were instructed to keep their hands on their waist, perform the CMJ with a knee angle of ~90°, and land with their legs extended with maximal plantar flexion. All participants performed 3 trials with 20 s (sec) of recovery between repetitions to minimize the effect of fatigue and three minutes between the different load jumps. The best jump in centimeters (cm) was considered the final outcome.

Repeated Jump 15″ (RJ15″): After performing the 505 test and following a 3 min rest period, the participants carried out one bout of maximal intensity (CMJs) for 15 s. Leg muscle power and jumping ability were assessed based on jump height. The same instrument used for CMJ measurements was employed. Based on this process, the maximum and minimum values were taken into account. Additionally, the fatigue index (FI) and reactive strength index (RSI) were calculated. FI was calculated by taking the average height (H) of the first 4 jumps and the average height of the last 4 jumps, as per Equation [47]:

$$Fatigue\;index=\frac{H\;first\;4\;jumps\;-\;H\;last\;4\;jumps}{H\;first\;4jumps\;}$$

Elsewhere, the RSI was calculated according to the following equation [48]:

$$RSI=\frac{Jump\;height\;(m)\;}{Ground\;Contact\;Time\;(s)}$$

Handgrip: Handgrip strength encompasses the maximum force generated through the combined contraction of the extrinsic and intrinsic muscles of the hand, resulting in the flexion of hand joints [49]. A hand dynamometer (TKK-5401, Takei Scientific Instruments, Niigata City, Japan) was employed to quantify this parameter. The players were seated in an upright position, facing the researcher, with their shoulder adducted and elbow flexed at a 90-degree angle while allowing the forearm to rest lightly on the arm of the chair or on the subject’s thigh. Alternating hand testing was conducted, with each hand undergoing two rounds of assessment interspersed with 10 s rest intervals [50].

COD 505 test: The methodology for the Chante of Direction 505 test (COD 5050 test) was originally established [51]. Therefore, this involved a 10 m linear sprint from a static start as well as a 5 m return through an identified finish line. The time between 5 m and turning the sprint line was recorded (seconds). All participants performed 2 attempts with 3 min of recovery between repetitions. The best time in seconds (sec) was recorded in a Microsoft Windows^®^ Excel template (Redmond, WA, USA). Chronojump Photocell^®^ (Chronojump, Barcelona, Spain) and Chronojump software version 1.7.1.8 were used to measure the time [52].

Yo-Yo test: YYIRT—Level 1 was used as a means of assessing participants’ aerobic capacity. The original protocol established by Krustrup et al. (2003) [53] was meticulously respected. The test consisted of performing a 2 × 20 m sprint, interspersed with a short 10 s walking recovery period. Starting at 10 km/h, determined by a beep, the intensity increased by 0.5 km/h until the player was exhausted. In the 10–13 km/h range, four 2 × 20 m sprints were performed, followed by seven runs at 13.5–14 km/h. Afterward, eight series were made for each stage [54]. The test was conducted within the confines of an indoor volleyball court. The conclusion of the test was determined when a player did not reach the required speed or did not reach the indicated line on the beep on two consecutive occasions. The total distance traveled during the test was recorded as the primary result.

### 2.4. Statistical Analysis

Descriptive statistics were calculated for each variable. The mean and standard deviation were used for data processing. Data normality was verified using the Shapiro–Wilk test, with parametric procedures of statistics, upon acceptance of normality presumptions. The experiment consisted of the within-participants factor of caffeine condition (caffeine condition and placebo condition) and moment condition (MD-4, MD-3, and MD-1). Internal load (sleep, fatigue, stress, and muscle soreness) and external load (handgrip dominant and non-dominant hand, COD 505 test, CMJ height, RJ height, RJ RSI, RJ min jump, RJ max jump, RJ fat index, and RJ time count) were analyzed using a repeated-measures ANOVA. The effect size is indicated with a partial eta squared for Fs. Posteriorly, a Pearson correlation coefficient r was used to examine the relationship between values of internal load and values of external load; to interpret the magnitude of these correlations, we adopted the following criteria: very small (0.01), small (0.20), medium (0.50), large (0.80), very large (1.20), and huge (2.0), as initially suggested by Cohen (1988) [55] and expanded by Sawilowsky (2009) [56]. The data were analyzed using the software Statistics (version 13.1; Statsoft, Inc., Tulsa, OK, USA), and the alpha level was set at *p* < 0.05.

## 3. Results

Descriptive statistics were calculated for each internal load (sleep, stress, fatigue, and muscle soreness) and external load (handgrip dominant and non-dominant hand, COD 505 test, CMJ height, RJ height, RJ RSI, RJ min jump, RJ max jump, RJ fat index, and RJ time count) variables. (See Table 1).

Different repeated measures ANOVA with participants’ mean sleep, fatigue, stress, and muscle soreness (internal load) were performed within-participants factor condition (caffeine condition and placebo condition) and moment condition (MD-4, MD-3, and MD-1). In the same vein, another similar analysis was performed with participants’ mean handgrip dominant and non-dominant, COD 505 test, CMJ height, RJ height, RJ RSI, RJ min jump, RJ max jump, RJ fat index, and RJ time count (external load) to try to elucidate the main effects and interactions of different measures (see Table 2 for more information).

Subsequently, different correlation analyses were performed between participants’ mean values of internal load (sleep, stress, fatigue, and muscle soreness) and external load (handgrip dominant and non-dominant, COD 505 test, CMJ height, RJ height, RJ RSI, RJ min jump, RJ max jump, RJ fat index, and RJ time count) in both conditions (caffeine and placebo). In this sense, large negative correlations were found between placebo RJ min jump and placebo stress (r = −0.75 and *p* = 0.02). Indeed, another correlation was found between RJ time con and placebo sleep (r = 0.72 and *p* = 0.03). No other correlations were found. (See Table 3 for more information.)

## 4. Discussion

This study aimed to observe the acute effect of caffeine intake over the course of one week of training in women’s semi-professional volleyball players. Regarding physical parameters, the caffeine condition obtained better results in handgrip, but only in the dominant hand. In a similar study, an enhancement of handgrip was found after ingestion of a drink containing 3 mg of caffeine per kilogram of body weight in women’s volleyball players [14]. Likewise, this finding was also found in men after a similar ingestion [4], so the results of handgrip improvement are similar in both men and women. This latter research also found similar results for improvements in CMJ after caffeine administration at a lower dose (3 mg/kg) in men’s volleyball players [4] and badminton players. Moreover, other studies reported an enhancement in CMJ with higher doses (≥6 mg/kg^−1^) in both women [39] and men [57].

Similarly, a subsequent study indicated that increased fiber recruitment via calcium release is associated with such high doses [58], indicating an improvement in isometric, concentric, and eccentric maximal voluntary contractions [59]. However, in this study, the ability to maintain this improvement over a week of training with an intake of 5 mg/kg body weight was also assessed. A main effect of supplementation was found over the training week. As mentioned above, volleyball is a sport where different jumps take place throughout the match, so it is essential to observe the effect of caffeine on repeated jumps, as the assessment of CMJ alone could not reveal a real match situation. Thus, previous studies have investigated the effect of caffeine in RJ, obtaining improvements in men’s volleyball players in RJ 15 s [4] and RJ 30 s [57]. It should be noted that no studies have been found on the effect of caffeine on RJ 15 s in women’s volleyball players. Therefore, this is the first study that evaluates this parameter in women, even though it is of vital importance, as previously explained. Additionally, it has been observed that when evaluating this parameter, there were improvements in the RSI and RJ min [57].

This is evidence that caffeine could produce better resistance to fatigue in terms of repeated jumps. This phenomenon could be due to several physiological mechanisms. Firstly, caffeine has been reported to produce hypoalgesia, whereby the decrease in pain inhibits the perception of overexertion and fatigue [60]. Alternatively, there is stimulation of the CNS through inhibition of the adenosine antagonist receptor, as well as increased production of catecholamines, epinephrine, and norepinephrine. Contradictorily, Karayigit et al. (2022) observed in women an increase in catecholamines after administration of 5.4 mg/kg body weight [61], but this was not reflected in the FI in repeated sprints nor in peak power, which was the case in the present investigation. However, the aforementioned study found an improvement in the mean power output of repeated sprints compared to a placebo. Another important aspect of caffeine on fatigue resistance is the increased production of lactate, which aids in the increased production of lactic anaerobic power [15,60,61,62]. Additionally, caffeine enhances sodium-potassium ATPase activity and intracellular calcium mobilization, indirectly affecting acetylcholine and dopamine release [60,61]. These facts could have helped to delay fatigue in repeated jumps. It should be noted that a recent review indicated that the effect of caffeine on jumping performance has the largest results in the follicular phase [63]. However, in the present investigation, only three of the eight participants were in this phase of the caffeine condition.

Caffeine attenuates the effects of fatigue by binding to adenosine receptors, reducing the RPE. However, the action of caffeine on the release and subsequent reuptake of calcium from the sarcoplasmic reticulum appears to be the reason for the attenuation of fatigue in short-duration, high-intensity tests [64]. This could be the reason for the improvement in fatigue in the COD 505 test, CMJ, RSI, and RJ minimum, which could also explain the improvement in perceived fatigue with caffeine in this study. Contradictorily, a recent meta-analysis [15] did not find an effect on RPE and agility. Similarly, a review conducted on football players also reported no statistical change in perceived fatigue in women [33]. A recent study reported an increased RPE with the administration of high doses of caffeine (>6 mg/kg) [58], while Del Coso et al. (2014) reported a lower RPE score (although not significant) in men’s volleyball players [4]. In addition, these authors documented greater insomnia when caffeine was consumed, in contrast to the present study, which did not find a decrease in sleep. Filip-Stachnik (2022) assessed sleep via actigraphy after caffeine intake (3 mg/kg) prior to an evening training session and found no sleep disturbance [39] as in this study. In this respect, the results are contradictory, as other previous studies have found a decrease in sleep. Miller et al. (2014) showed a decrease in sleep efficiency in triathletes after administration of two doses of 3 mg/kg [65]. A subsequent study found similar results in 800 m athletes with the administration of 6 mg/kg caffeine [37]. It has been suggested that the differences found in the studies could be due to the difference in dose due to the stimulation of catecholamines and a decrease in 6-sulphatoxymelatonin [39]. However, in the present study, no differences were found after an administration of 5 mg/kg. It should be noted that five of the eight players were in the luteal phase in the caffeine condition, so it could influence the results. However, a similar study suggested that the results were not affected by menstrual cycle phases [14]. It has been suggested that the pharmacokinetics of caffeine could be affected by altering the stimulant effect of caffeine [66]. Specifically, in the follicular phase, caffeine metabolism could be slower due to estradiol [67]. In addition, estradiol appears to play an important role in muscle protection against exercise stress, showing signs of reducing the inflammatory response observed in women. This protective function of estradiol appears to be a key factor in decreasing exercise-associated muscle damage [68,69]. Alternatively, the high estrogen concentration at the onset of the luteal phase could influence higher fatigue tolerance in the participants [70]. Nevertheless, recent research results indicate that caffeine is ergogenic in all phases of the menstrual cycle [71,72].

Concerning muscle damage, a main effect of caffeine was found in relation to perceived muscle damage (*F* (1.7) = 7.29, *p* = 0.03, and η2 = 0.51). Accordingly, it has been suggested that pre-exercise caffeine intake could improve perceived muscle damage [40]. In this regard, a meta-analysis revealed that caffeine decreased muscle damage after 48 h post-exercise compared to a placebo [73]. Thus, in this study, the participants trained on Tuesdays, Thursdays, and Fridays, with the results coinciding with the aforementioned study. This could be because caffeine could improve peripheral neuromuscular transmission [74]. Caffeine would delay the failure of postsynaptic transmission [75], as well as the decrease in membrane action potentials [76], and inhibition of the central nervous system on motor neurons [77].

This study has some limitations. Firstly, the number of the sample size is one limitation, although a sample calculation was previously carried out in a similar study. The power analysis conducted in the aforementioned study determined that a minimum sample size of 5 athletes was necessary [45]. Secondly, although the players were urged not to consume caffeine on measurement days, they were regular coffee drinkers (<100 mg/day), which could affect the results of the study. However, recent research found that regular caffeine consumption did not interfere with the potential of caffeine as an ergogenic aid in explosive exercise enhancement [78]. Similarly, a recent studies reported that caffeine intake of 3–6 mg/kg improved 1 RM in women habituated to caffeine ingestion [79,80]. Finally, the menstrual cycle of the women was calculated from the last date of menstruation instead of measuring them with more accurate methods. In addition, the fact that the participants were at different stages in the two conditions is another factor that could possibly have influenced the results. However, several studies have reported that caffeine has an ergogenic effect in all phases of the menstrual cycle [41,70,71]. Lastly, although an initial nutritional survey was conducted, the diet was not controlled throughout the investigation, so nutritional intake may have influenced the results.

## 5. Conclusions

It appears that administration of 5 mg/kg body weight increases CMJ, RSI, and handgrip in semi-professional women’s volleyball players over one week of training. Additionally, it seems that caffeine supplementation enhances RSI and FI. Moreover, this ergogenic aid improves the perception of fatigue. Further similar research is needed in women that collects both physical performance and well-being parameters and correlates these variables, particularly in highly competitive periods such as the playoffs.

## Figures and Tables

**Table 1:** Within-week variations (MD-4, MD-3, and MD-1) of (i) internal load: sleep, stress, fatigue, and muscle soreness and (ii) external load: handgrip dominant and non-dominant hand, COD 505 test, CMJ height, RJ height, RJ RSI, RJ min jump, RJ max jump, RJ fat index, and RJ time count (mean ± SD).

	Caffeine Condition			Placebo Condition		
	MD-4	MD-3	MD-1	MD-4	MD-3	MD-1
**Internal load**						
**Sleep (AU)**	3.38 ± 1.92	2.88 ± 2.10	2.88± 1.81	4.38 ±1.77	3.50 ± 1.51	2.88 ± 1.81
**Fatigue (AU)**	3.00 ± 1.51	3.13 ± 1.36	3.25 ± 2.19	4.00 ± 1.60	3.63 ± 1.60	3.50 ± 1.93
**Stress (AU)**	3.75 ± 1.28	3.50 ± 1.85	2.88 ± 1.73	3.88 ± 0.99	4.25 ± 1.39	2.88 ± 1.13
**Muscle soreness (AU)**	2.63 ± 1.19	3.13 ± 1.55	3.50 ± 2.33	3.25 ± 1.04	3.88 ± 1.46	4.50 ± 1.41
**External load**						
**Handgrip dominant (kg)**	33.61 ± 4.10	35.58 ± 3.71	35.04 ± 3.46	34.46 ± 3.73	33.19 ± 3.21	35.35 ± 4.71
**Handgrip non-dominant (kg)**	31.83 ± 4.39	32.51 ± 4.16	31.01 ± 4.63	32.62 ± 2.08	32.23 ± 4.47	32.60 ± 3.89
**COD 505 test (s)**	4.32 ± 0.19	4.17 ± 0.19	4.13 ± 0.14	4.31 ± 0.23	4.17 ± 0.19	4.15 ± 0.11
**CMJ height (cm)**	34.18 ± 5.60	37.18 ± 4.70	35.47 ± 6.09	30.69 ± 4.42	34.72 ± 5.95	33.88 ± 6.99
**RJ height (cm)**	28.27 ± 2.76	30.28 ± 2.40	30.29 ± 3.41	26.18 ± 2.61	28.54 ± 4.53	27.79 ± 2.98
**RJ RSI (m/s)**	1.18 ± 0.16	1.35 ± 0.25	1.35 ± 0.26	1.02 ± 0.16	1.26 ± 0.17	1.19 ± 0.15
**RJ min jump (cm)**	20.32 ± 2.36	24.73 ± 2.58	23.07 ± 2.68	19.45 ± 3.68	24.25 ± 5.30	24.48 ± 4.37
**RJ max jump (cm)**	34.16 ± 2.09	32.77 ± 3.63	32.09 ± 3.79	31.33 ± 3.71	32.64 ± 3.75	32.53 ± 3.68
**RJ FI (%)**	108.70 ± 14.40	101.98 ± 12.02	97.75 ± 9.69	94.98 ± 12.85	96.25 ± 11.51	94.60 ± 11.47
**RJ contact time (s)**	0.34 ± 0.11	0.25 ± 0.04	0.23 ± 0.03	0.32 ± 0.11	0.25 ± 0.06	0.25 ± 0.05

**Note**—AU: Arbitrary Units; COD: Change of Direction; CMJ: Countermovement Jump; RJ: Repeat Jump; RSI: Relative Strength Index; FI: Fatigue Index; Min: Minimum, and Max: Maximum.

**Table 2:** Effect of condition, moment, and interaction condition. * Moment for internal load and external load (mean ± SD).

	Main Effect of Condition	Main Effect of Moment	Interaction Condition * Moment
**Internal Load**			
**sleep (AU)**	*F* (1.7) = 2.85, *p* = 0.13, η2 = 0.28	*F* (2.14) = 2.88, *p* = 0.09, η2 = 0.29	*F* < 1
**Fatigue (AU)**	*F* (1.7) = 7.29, *p* = 0.03 *, η2 = 0.51	*F* < 1	*F* (2.14) = 1.04, *p* = 0.37, η2 = 0.1
**Stress (AU)**	*F* < 1	*F* (2.14) = 4.69, *p* = 0.02 *, η2 = 0.40	*F* (2.14) = 1.58, *p* = 0.23, η2 = 0.18
**Muscle soreness (AU)**	*F* (1.7) = 7.54, *p* = 0.02 *, η2 = 0.52	(2.14) = 2.46, *p* = 0.12, η2 = 0.26	*F* < 1
**External Load**			
**Handgrip dominant (kg)**	*F* (1.7) = 1.18, *p* = 0.32, η2 = 0.19	*F* < 1	*F* (2.14) = 9.56, *p* = 0.004 **, η2 = 0.65
**Handgrip non-dominant (kg)**	*F* < 1	*F* < 1	*F* < 1
**COD 505 test (sec)**	*F* < 1	*F* (2.14) = 4.61, *p* = 0.03 *, η2 = 0.39	*F* < 1
**CMJ height (cm)**	*F* (1.7) = 8.41, *p* = 0.02 *, η2 = 0.54	*F* (2.14) = 6.40, *p* = 0.01 *, η2 = 0.47,	*F* < 1
**RJ height (cm)**	*F* (1.7) = 5.97, *p* = 0.04 *, η2 = 0.46	*F* (2.14) = 8.57, *p* = 0.001 **, η2 = 0.55	*F* < 1
**RJ RSI (m/s)**	*F* (1.7) = 22.88, *p* = 0.001 **, η2 = 0.76	*F* (2.14) = 12.91, *p* = 0.001 **, η2 = 0.64	*F* < 1
**RJ min jump (cm)**	*F* < 1	*F* (2.14) = 15.18, *p* = 0.001 **, η2 = 0.68	*F* < 1
**RJ max jump (cm)**	*F* (1.7) = 1.53, *p* = 0.25, η2 = 0.17	*F* < 1	*F* (2.14) = 1.85, *p* = 0.19, η2 = 0.29
**RJ FI (%)**	*F* (1.7) = 7.33, *p* = 0.03 *, η2 = 0.51	*F* (2.14) = 1.20, *p* = 0.32, η2 = 0.14,	*F* < 1
**RJ contact time (s)**	*F* < 1	*F* (2.14) = 20.81, *p* = 0.001 **, η2 = 0.74	*F* < 1

**Note**—AU: Arbitrary Units; COD: Change of Direction; CMJ: Countermovement Jump; RJ: Repeat Jump; RSI: Relative Strength Index; FI: Fatigue Index; Min: Minimum, and Max: Maximum. * Denotes significance at *p* < 0.05, and ** denotes significance at *p* < 0.01.

**Table 3:** Pearson’s correlation coefficient between (i) internal load: sleep, stress, fatigue, and muscle soreness and (ii) external load: handgrip dominant and non-dominant, COD 505 test, CMJ height, RJ height, RJ RSI, RJ min jump, RJ max jump, RJ fat index, and RJ time count (mean ± SD).

	Caffeine Sleep	Placebo Sleep	Caffeine Fatigue	Placebo Fatigue	Caffeine Stress	Placebo Stress	Caffeine MS	Placebo MS
**Caffeine HG Dom**	r = 0.12	r = −0.01	r = 0.13	r = −0.12	r = 0.12	r = −0.57	r = 0.01	r = −0.13
	*p* = 0.76	*p* = 0.98	*p* = 0.73	*p* = 0.74	*p* = 0.75	*p* = 0.10	*p* = 0.97	*p* = 0.73
**Placebo HG Dom**	r = −0.17	r = −0.17	r = 0.11	r = −0.05	r = 0.14	r = −0.59	r = 0.14	r = 0.02
	*p* = 0.65	*p* = 0.65	*p* = 0.76	*p* = 0.89	*p* = 0.70	*p* = 0.09	*p* = 0.70	*p* = 0.94
**Caffeine HG Non-Dom**	r = −0.38	r = −0.29	r = 0.06	r = 0.11	r = 0.25	r = −0.48	r = 0.32	r = 0.16
	*p* = 0.31	*p* = 0.43	*p* = 0.87	*p* = 0.76	*p* = 0.50	*p* = 0.18	*p* = 0.38	*p* = 0.67
**Placebo HG Non-Dom**	r = −0.01	r = −0.03	r = 0.27	r = −0.13	r = 0.12	r = −0.57	r = 0.01	r = −0.17
	*p* = 0.99	*p* = 0.93	*p* = 0.47	*p* = 0.75	*p* = 0.75	*p* = 0.10	*p* = 0.97	*p* = 0.66
**Caffeine COD 505 test**	r = 0.17	r = −0.10	r = 0.08	r = 0.20	r = 0.11	r = 0.06	r = 0.23	r = 0.44
	*p* = 0.65	*p* = 0.78	*p* = 0.83	*p* = 0.59	*p* = 0.76	*p* = 0.85	*p* = 0.54	*p* = 0.22
**Placebo COD 505 test**	r = 0.15	r = −0.17	r = 0.09	r = 0.32	r = 0.15	r = 0.26	r = 0.29	r = 0.50
	*p* = 0.69	*p* = 0.65	*p* = 0.79	*p* = 0.39	*p* = 0.68	*p* = 0.48	*p* = 0.44	*p* = 0.17
**Caffeine CMJ height**	r = −0.01	r = 0.25	r = 0.03	r = 0.01	r = −0.04	r = 0.16	r = 0.03	r = −0.22
	*p* = 0.96	*p* = 0.51	*p* = 0.93	*p* = 0.98	*p* = 0.90	*p* = 0.67	*p* = 0.93	*p* = 0.55
**Placebo CMJ height**	r = −0.04	r = 0.32	r = −0.04	r = −0.21	r = −0.16	r = −0.08	r = −0.14	r = −0.43
	*p* = 0.89	*p* = 0.39	*p* = 0.90	*p* = 0.58	*p* = 0.67	*p* = 0.82	*p* = 0.71	*p* = 0.24
**Caffeine RJ height**	r = −0.09	r = −0.26	r = −0.14	r = 0.06	r = 0.04	r = 0.08	r = 0.02	r = 0.45
	*p* = 0.80	*p* = 0.49	*p* = 0.70	*p* = 0.87	*p* =0.91	*p* = 0.83	*p* = 0.95	*p* = 0.21
**Placebo RJ height**	r = −0.13	r = −0.37	r = −0.26	r = −0.06	r = 0.08	r = 0.40	r = −0.05	r = 0.28
	*p* = 0.73	*p* = 0.32	*p* = 0.49	*p* = 0.86	*p* = 0.83	*p* = 0.28	*p* = 0.88	*p* = 46
**Caffeine RJ RSI**	r = −0.19	r = −0.13	r = −0.05	r = 0.01	r = −0.09	r = −0.34	r = 0.17	r = 0.17
	*p* = 0.62	*p* = 0.73	*p* = 0.88	*p* = 0.98	*p* = 0.81	*p* = 0.36	*p* = 0.65	*p* = 0.65
**Placebo RJ RSI**	r = 0.06	r = 0.18	r = 0.07	r = 0.04	r = −0.05	r = −0.24	r = 0.24	r = 0.12
	*p* = 0.86	*p* = 0.62	*p* = 0.84	*p* = 0.91	*p* = 0.88	*p* = 0.53	*p* = 0.52	*p* = 0.75
**Caffeine RJ min jump**	r = 0.09	r = 0.17	r = 0.15	r = 0.11	r = 0.16	r = −0.21	r = 0.05	r = 0.32
	*p* = 0.79	*p* = 0.66	*p* = 0.69	*p* = 0.77	*p* = 0.67	*p* = 0.57	*p* = 0.88	*p* = 0.39
**Placebo RJ min jump**	r = −0.20	r = 0.07	r = −0.27	r = −0.51	r = −0.49	r = −0.75	r = −0.41	r = −0.52
	*p* = 0.59	*p* = 0.85	*p* = 0.47	*p* = 0.15	*p* = 0.17	*p* = 0.02 *	*p* = 0.26	*p* = 0.14
**Caffeine RJ max jump**	r = 0.18	r = 0.44	r = 0.13	r = 0.02	r = −0.20	r = −0.36	r = 0.07	r = −0.09
	*p* = 0.63	*p* = 0.22	*p* = 0.72	*p* = 0.94	*p* = 0.60	*p* = 0.33	*p* = 0.85	*p* = 0.81
**Placebo RJ max jump**	r = 0.24	r = 0.38	r = −0.10	r = −0.29	r = −0.33	r = −0.22	r = −0.22	r = −0.30
	*p* = 0.53	*p* = 0.31	*p* = 0.79	*p* = 0.43	*p* = 0.37	*p* = 0.55	*p* = 0.56	*p* = 0.42
**Caffeine RJ FI**	r = −0.27	r = −0.42	r = −0.32	r = −0.11	r = −0.17	r = 0.08	r = −0.34	r = 0.07
	*p* = 0.47	*p* = 0.24	*p* = 0.38	*p* = 0.76	*p* = 0.65	*p* = 0.82	*p* = 0.35	*p* = 0.84
**Placebo RJ FI**	r = 0.18	r = 0.07	r = 0.26	r = 0.41	r = 0.28	r = 0.54	r = 0.13	r = 0.17
	*p* = 0.63	*p* = 0.84	*p* = 0.49	*p* = 0.27	*p* = 0.45	*p* = 0.13	*p* = 0.73	*p* = 0.64
**Caffeine RJ time**	r = 0.60	r = 0.72	r = 0.46	r = 0.36	r = 0.27	r = 0.40	r = 0.20	r = 0.09
	*p* = 0.08	*p* = 0.03 *	*p* = 0.20	*p* = 0.33	*p* = 0.47	*p* = 0.28	*p* = 0.59	*p* = 0.81
**Placebo RJ time**	r = −0.22	r = −0.38	r = −0.45	r = −0.46	r = −0.25	r = 0.15	r = −0.53	r = −0.37
	*p* = 0.55	*p* = 0.30	*p* = 0.21	*p* = 0.20	*p* = 0.51	*p* = 0.70	*p* = 0.13	*p* = 0.31

**Note**—COD: Change of Direction; CMJ: Countermovement Jump; RJ: Repeat Jump; RSI: Relative Strength Index; FI: Fatigue Index; HG: Handgrip; Dom: Dominant; Non-Dom: Non-Dominant; and MS: Muscle Soreness. * Significance at *p* < 0.05.

## Data Availability

Data are contained within the article.
